# Implementing artificial neural networks through bionic construction

**DOI:** 10.1371/journal.pone.0212368

**Published:** 2019-02-22

**Authors:** Hu He, Xu Yang, Zhiheng Xu, Ning Deng, Yingjie Shang, Guo Liu, Mengyao Ji, Wenhao Zheng, Jinfeng Zhao, Liya Dong

**Affiliations:** 1 Institute of Microelectronics, Tsinghua University, Beijing, China; 2 School of Computer Science and Technology, Beijing Institute of Technology, Beijing, China; Newcastle University, UNITED KINGDOM

## Abstract

It is evident through biology research that, biological neural network could be implemented through two means: by congenital heredity, or by posteriority learning. However, traditionally, artificial neural network, especially the Deep learning Neural Networks (DNNs) are implemented only through exhaustive training and learning. Fixed structure is built, and then parameters are trained through huge amount of data. In this way, there are a lot of redundancies in the implemented artificial neural network. This redundancy not only requires more effort to train the network, but also costs more computing resources when used. In this paper, we proposed a bionic way to implement artificial neural network through construction rather than training and learning. The hierarchy of the neural network is designed according to analysis of the required functionality, and then module design is carried out to form each hierarchy. We choose the Drosophila’s visual neural network as a test case to verify our method’s validation. The results show that the bionic artificial neural network built through our method could work as a bionic compound eye, which can achieve the detection of the object and their movement, and the results are better on some properties, compared with the Drosophila’s biological compound eyes.

## Introduction

Researches about artificial intelligence have become very popular in current days, due to the ever growing demands from application domains such as pattern recognition, image segmentation, intelligent video analytics, and autonomous robotics [1–4]. The problems in artificial intelligence domain are mostly unstructured problems. Unlike structured problems, which could break down into a series of well-defined steps, and represent precisely by mathematical formulas, solving of unstructured problems requires the use of intuition, reasoning, and memory. Artificial neural network is more suitable for solving unstructured problems than traditional von Neumann architecture, thus becomes the core part of artificial intelligence research.

Nowadays, Deep Neural Network (DNN) has become the research hot-spot of artificial neural networks [5], because it has won plentiful contests against people, including the most famous one recently, Google’s AlphaGo DLNN beating Lee Sedol, a famous I-go master. However, DNN has some disadvantages:

1. apt to be cheated when trained with small scale of data [6]. Since DNN typically exploits rather fixed network structure, and could not change the structure to reflex the changing in the environment, there would be a lot of redundant parameters, making the training result less optimized when a merely small scale of data been feed, and apt to be deceived [6].
2. demanding a huge amount of computing power when trained with a large scale of data. When trained with large scale of data, DNN becomes more accurate. However, huge amount of computing power is needed to accomplish the training process. This will become a bottleneck when the scale of data becomes even larger.
3. could deal with classification or regression, but not good at doing intuition or reasoning. Since there are no feedback loops in DNN, it can only process data stream in an unidirectional way, meaning the comparison of data for the past and now is not possible. So, it is unfeasible to accomplish intuition or reasoning using DNN.

This is mainly due to the fact that the traditional ANN, including DNN, uses essentially the same and fixed structure even for different problems. This can lead to a large number of redundancy in the network:

1. Redundant structure and parameter training consumes extra energy and effort, and lengthens the training time;
2. Redundant structures can lead to additional energy and computational power to be consumed during the use of the network.

It is evident through biology research that, biological neural network could be implemented through two means: by congenital heredity, or by posteriority learning. Training and learning process could implement powerful artificial neural network. However, in biological neural systems, some neural network structures are just inherited directly from their parents, and implemented through construction only. Those kinds of structure usually are used to perform specific functionality, and have a more compact and concise topology.

In this paper, we propose a way to implement artificial neural network through bionic construction rather than training and learning. Hierarchy of the neural network will be designed according to analysis of the required functionality, and then the module design is carried out to form the hierarchy. We choose Drosophila’s visual neural network as a test case to testify our method’s validation.

The following is organized as: Section 2 discusses related work; Section 3 presents our method; Section 4 discusses the structure of Drosophila’s visual system briefly; Section 5 gives details of our test case research and the experiment result; finally we give the conclusion.

## Related work

In recent years, many researchers have shown their interests on constructing the bionic artificial neural network. Zhang et al. have constructed a bionic neural network based on the study of the olfactory neural system, which exhibits chaotic characteristics and has potential on pattern recognition [7]. Li has designed a novel hierarchical modular echo state network(HMESN) based on brain networks, in which each level of neurons using small world network construction algorithm to generate the modular structure. Based on the topological characteristics of hierarchical modular in brain network, it weakens the coupling intensity among neurons and enriches the dynamics of internal neurons [8].

As a prime model for biological study, Drosophila has received many research interests. Chang et. al. [9] have examined the robustness of a simplified neural circuitry built from about ten thousand single neurons of the Drosophila brain. They found the network is resilient under both errors and attacks, and they have observed how such a resiliency is associated with the accumulation of neurons along their birth stages. Lin et. al. [10] have analyzed the neurons in the Drosophila brain, found the possible connecting path, and furthermore, performed cluster analysis from the connectivity information of thousands of tangled neurons. Arena et. al. [11] have proposed a new model of the olfactory system of the fruit fly Drosophila melanogaster. The architecture is a multi-layer spiking neural network, inspired by the structures of the insect brain mainly involved in the olfactory conditioning, namely the Mushroom Bodies, the Lateral Horns and the Antennal Lobes. Later they have also proposed a neural-based model for the formation of a spatial working memory mirroring some peculiarities of the Drosophila central brain and in particular the ellipsoid body [12].

Akhmedova et al. have presented an artificial neural network with Co-Operation of Biology Related Algorithms (COBRA) to solve multi-objective unconstrained problems. Their experiment results showed that both variants of COBRA demonstrate high performance and reliability in spite of the complexity of the optimization problems solved [13]. Chen et al. have presented a bio-inspired neural network for improvement of information processing capability of the existing artificial neural networks. They introduced a property often found in biological neural system—hysteresis—as the neuron activation function and a bionic algorithm [14]. Hu et al. have presented their effort on constructing auto-grow and auto-evolve bionic artificial neural network. They have developed a paralleled simulation platform, and have explored ways to promote the neural network to auto-generate as a response to external pulses [15, 16].

## Bionic construction method to implement artificial neural network

According to biology discoveries, there are mainly two kinds of neural network structures:

1. Learning-type neural network: Usually can simultaneously perform multiple diverse functionalities, with a more generalized topology, and could be optimized by training and learning;
2. Constructing-type neural network: Usually developed for one or several specific functionality, with a more compact, concise, and distinctive topology, and could be optimized through evolution between generations.

Traditionally, no matter what kind of functionalities needs to achieve, the researchers tend to use learning-type neural network, that is to say, implement artificial neural networks through training or learning. However, by comparison of learning-type neural network and constructing-type neural network in biology systems, we could conclude that they are obviously different in topology. If we use learning-type neural network to perform some functionalities that are more suitable for constructing-type neural network, there will be a lot of redundancies in the neural network. That will harm the neural network in many ways, like the training effort or the running efficiency.

**Fig 1:**
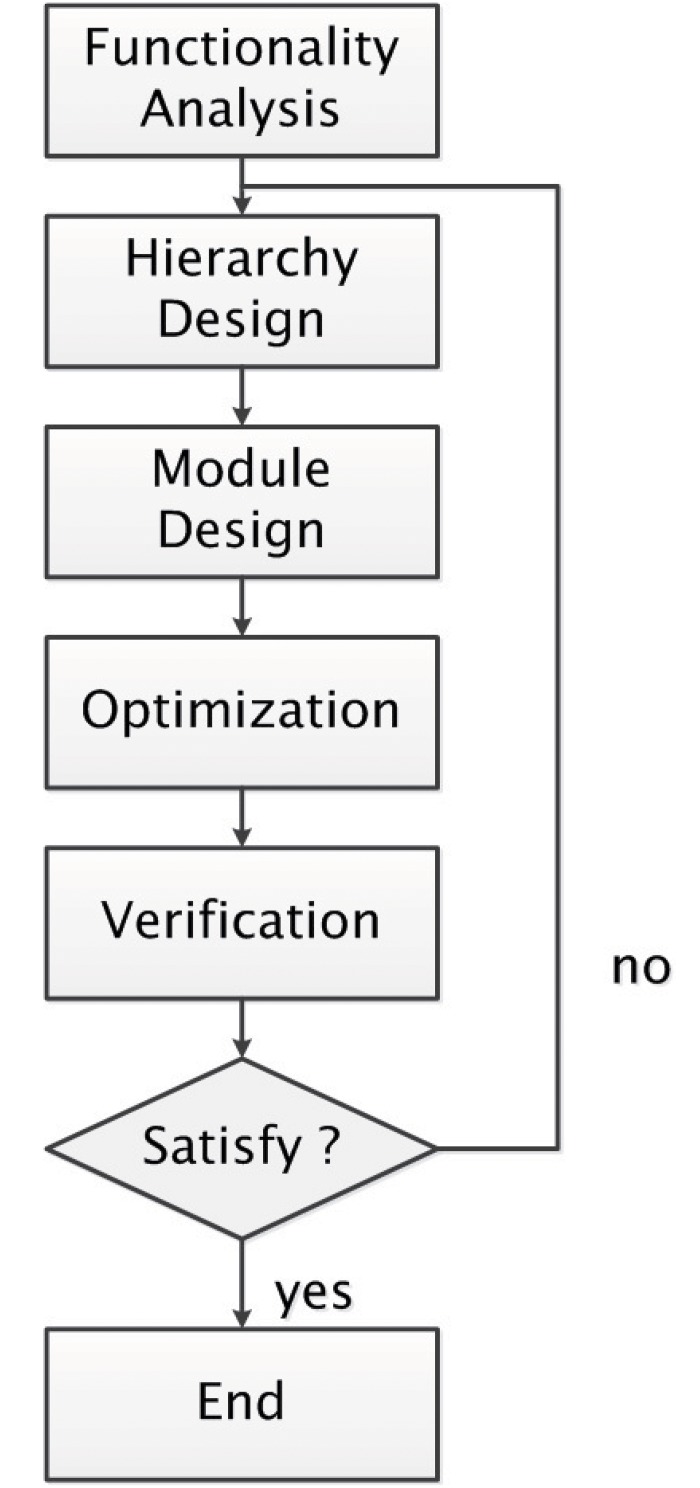
Flow of our method.

In this section, we propose a method to implement neural network through bionic construction. The flow of this method is shown in Fig 1.

The method proposed is used to guide the implementing of constructing-type neural network, so the functionality is usually distinctive. The purpose of functionality analysis is to define the form of the input stimulus and output of the neural network, and find a possible solution of hierarchy design for the target neural network. During hierarchy design phase, for each hierarchy, its distinct function is defined, and input stimulus and output form decided. Then, through module design, the module (the smallest part of a hierarchy, repeat it a lot of time to construct a hierarchy) is designed, sometimes needs evolution process. After that, an optimization phase is carried out, to remove redundancy from the neural network, and check for any part that could be improved. A verification phase is needed to find out if the current neural network satisfies the required functionality. If not, this process needs repeated again.

We will use Drosophila’s visual neural system as a test case, to show how our method works, and to testify its validation.

## Drosophila’s visual system

A compact genome and a tiny brain make Drosophila the prime model to understand the neural substrate of behavior [17]. Its visual neural network has distinguished and regular characteristics, making it suitable for bionic research. Currently, the research of Drosophila’s visual neural network focuses on motion analysis, color recognition, and pattern recognition.

Drosophila’s visual neural network is consisted of four levels: retina, stroma, medulla, and lobule & lobular plate. The retina, an optically compound eye, is composed of regularly arranged ommatidia, each of which contains eight photo-receptors (R1-R8) in addition to supporting cells, to detect light ranging from UV to green [18]. The retina is in charge of receiving external signals and propagating signals through the network. The stroma handles the detection of motion, and the medulla deals with delay. Based on the pulse that previous levels generated, the lobule & lobular plate gives the decision and promotes decisions to the brain.

Although many of the details of how this system works are unknown to us, we know that Drosophila’s visual system has following features:

1. more sensitive to transverse motion, less sensitive to vertical motion
2. less effective to detect static object
3. unable to see far away object

## Case study

### Functionality analysis

Our target is to form an artificial neural network that could detect a moving object’s velocity. In order to achieve this, we need to:

1. Able to sense that moving object;
2. Know the moving object’s position;
3. Know the moving object’s current position at any time;
4. Build its moving track, and identify its moving direction;
5. Calculate its velocity.

So, we could design the hierarchy of the neural network according to this analysis.

### Hierarchy design

The reason to design the hierarchy is to make the separate constructing for each layer possible. So, each layer should have a specific and unique function, which means we can decide the hierarchy according to the function division.

The goal for this bionic artificial neural network is to decide a moving object’s velocity, so we can build the hierarchy like this:

1. a layer to give the final result, as the velocity of that moving object
2. a layer to identify the moving track of that object, and exact time when that object is at a certain location. With that information, we are able to calculate the velocity.
3. a layer to identify the current location of that object. With this layer, we can know the information of the location and time pair for that moving object, then propagate that information for the upper layer to form the moving track.
4. a layer to identify the current perception area of that object. In Drosophila’s compound eye, there are regularly arranged ommatidia, each of which contains eight photo-receptors. So, when a moving object is before a Drosophila, more than one ommatidia would perceive it. We need information of which ommatidia have currently perceived the moving object to decide the location of that object.
5. a layer to perceive the moving object.

So, we decide our neural network should consist of five layers: perception layer, range estimation layer, location identification layer, track recognition layer and final layer.

### Module design

We will discuss the design of modules in each layer in detail in this part.

#### Perception layer

This layer is designed to perceive any object. According to research, Drosophila has a fixed angle of view. In this work, we set this angle of view as 120 degree, which means each of the perceive neurons can perceive the area of a cone with a 120-degree angle. We arrange all the perceive neurons on a plane.

**Fig 2:**
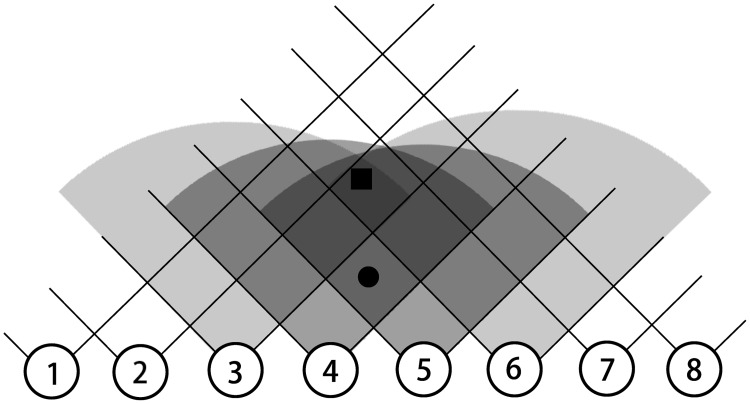
1-dimensional perception layer.

Let’s explain how this perception layer works first using a one-dimensional example as shown in Fig 2. There are two objects in this figure, shown as one black rectangle and one black circle, respectively. The black circle could be perceived by perceiving neuron 4, and 5, while the black rectangle could be observed by perceiving neuron 3, 4, 5, and 6. If a neuron *PL*_*i*_ in perception layer observes an object, it would output a spiking signal to the next layer. So the model of neurons *PL*_*i*_ in perception layer can be described as:

$$O_{PL_{i}}=\{\begin{array}{c} 1,if\;neuron\;i\;observes\;an\;object;\\ 0,if\;neuron\;i\;observes\;nothing; \end{array}$$ (1)

**Fig 3:**
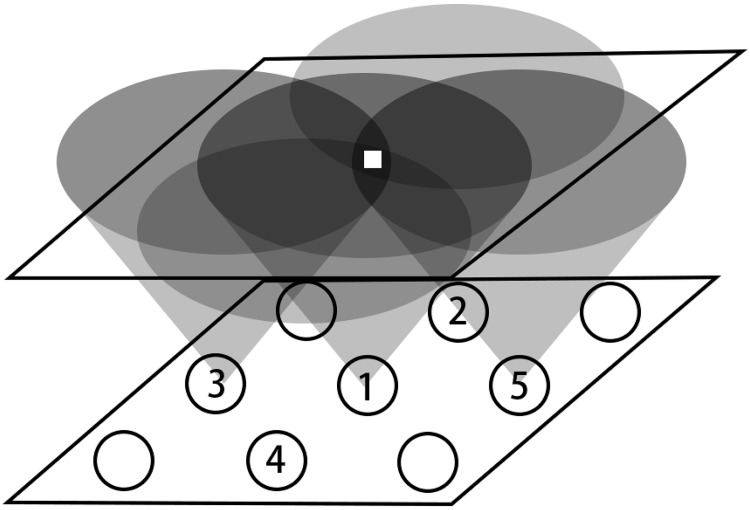
2-dimensional perception layer.

Fig 3 shows how it works in the two-dimensional network. If an object is in the location of the black circle shown in the figure, then it would be perceived by perceiving neuron 1, 2, 3, 4, and 5.

When the object is moving in a transverse direction, then with its moving, different perceive neurons would perceive it in a different time, thus could help us to determine its moving track.

When the object is moving in a vertical way, if it gets nearer to the plane, then, less and less perceive neurons would perceive it, while if the object is moving away from the plane, then more and more perceive neurons will be able to perceive it. This information could be used to estimate the distance of that object from the plane.

However, if the object is far enough from the plane, then all the perceive neurons would perceive it, and the distance from that object to different perceive neurons would be almost the same, making the perception layer less sensitive to its moving, just like the third feature of Drosophila’s visual neural network: unable to see far away object.

#### Range estimation layer

The information collected by the perception layer would be propagated to this layer to find out the perceived range for the object. Each neuron in range estimation layer connects to an area of neurons in the perception layer. The shape and size of the area can be set according to the requirements for the recognition ability of neurons in this layer.

When an object appears within the range of the perception layer connected by a neuron in the range estimation layer, the neuron receives spiking signals from one or more neurons in the perception layer. In order to gain more specific information about that object, such as the exact location, the connections between neurons from perception layer and the neuron in the range estimation layer are assigned different weight.

**Fig 4:**
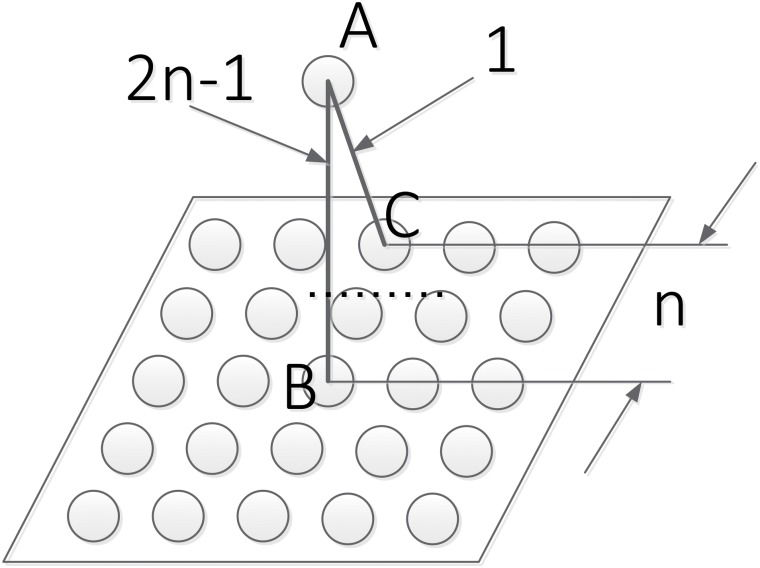
Different weight for different connections.

As shown in Fig 4, the plane is the perception layer, and the single neuron (call it neuron A) is one neuron in range estimation layer. If the distance between the perceive neuron that directly below neuron A (call it neuron B) and the out most perceive neuron (call it neuron C) that has a connection with neuron A is *n*, then, the weight of the connection between neuron A and neuron B is 2*n* − 1, while the weight of the connection between neuron A and neuron C is 1. The weight for the connection of those between them can be calculated accordingly.

**Fig 5:**
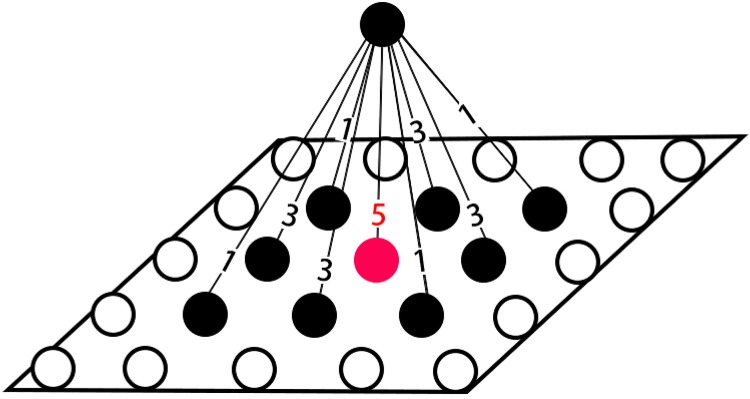
Connection between layer 1 to layer 2.

Fig 5 gives an example when *n* is 3.

This paper presents an available method to set the weight, does not necessarily mean that this is the optimal weight distribution, but because of the weight distribution, which is easy to expand the network. We choose this weight assignment mechanism for mainly two reasons:

1. Convenient when extending the network. When the network scale is enlarged, a simple method of weight calculation can be used, so it can be easily applied to any network of any size.
2. Under this weight assignment mechanism, only one maximum value can be obtained by the second layer neurons’ calculation, which is convenient for the posterior layer neuron to make decision.

The neurons in range estimation layer would calculate the weighted spiking signals it receives from perception layer to find out useful information about the distance and location of the moving object. So the model of neurons in the range estimation layer can be described by Eq (2):

$$O_{REL_{j}}=\sum w_{ij}O_{PL_{i}}$$ (2)

Where $O_{REL_{j}}$ is the output of neuron *j* in the range estimation layer; $O_{PL_{i}}$ is output of neuron *i* in the perception layer; *w*_*ij*_ is the weight of that connection between those two neurons. The sum counts for all the connections from perception layer to neuron A.

#### Location identification layer

Range estimation layer propagates the sum it generates to the location identification layer. Each neuron in the location identification layer connects to five neurons in the previous layer and compares the output values of those neurons. If it finds out that the neuron that directly before it outputs the maximum value, then a spiking is generated by this neuron and sent to the next layer to identify that the object is currently in its location. The model of neurons in location identification layer can be expressed as Eq (3)

$$O_{LIL_{i}}=\{\begin{array}{cc} 1 & if\;O_{REL_{i}}=MAX\{O_{REL_{1}},...,O_{REL_{m}}\}\\ 0 & if\;O_{REL_{i}}<MAX\{O_{REL_{1}},...,O_{REL_{m}}\} \end{array}$$ (3)

Where $O_{LIL_{i}}$ is the output of neuron *i* of the location identification layer; $O_{REL_{i}}$ is output of neuron *i* of the range estimation layer (Here it has the same label *i* as the previous neuron, meaning it is directly before that neuron).

#### Track recognition layer

Location identification layer identifies the location of the object. If that object is moving, then different neurons in location identification layer would give out spiking signals on different time. Track recognition layer uses this information to track the object and gives the direction through spiking signal to next layer.

**Fig 6:**
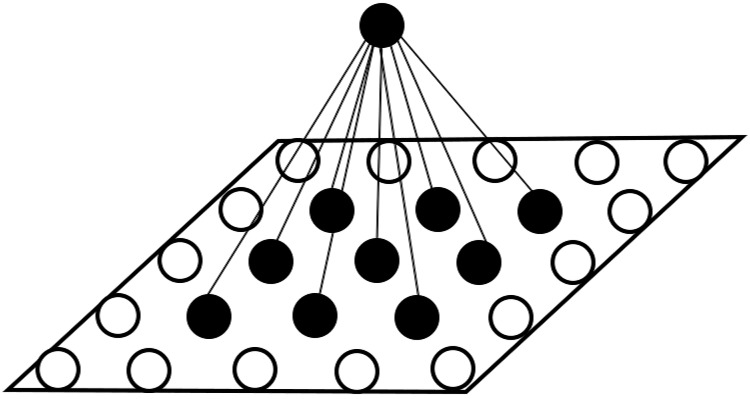
Connection between layer 3 to layer 4.

The connection between the neurons in the fourth layer and the neurons in the third layer is shown in Fig 6. This layer requires two cycles of detection to complete its task. When a neuron in the fourth layer receives a spiking signal from the neuron that directly before it in the third layer(in Fig 6, it’s the neuron that directly below it), this neuron will be activated, thus the detection mechanism turns on.

**Fig 7:**
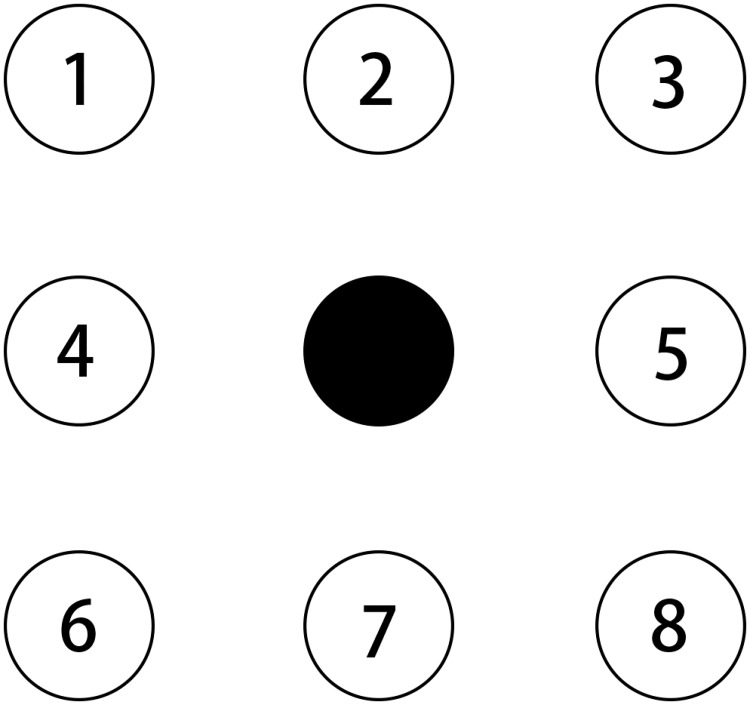
The orientation information of the output.

If at the next moment spiking signal appears in the upper left corner, then the output value of the fourth layer for the direction of motion is 1. The output is shown in Fig 7. A total of eight directions to determine the output value, respectively, the output value from 1 to 8. Further, if the output direction determination is not zero (i.e., a number between 1-8), the neuron of the fourth layer sends a single spiking signal to the next layer. When the first spiking signal passes through the eight neurons, a preliminary information of the direction is obtained, and when the second spiking signal arrives, the information can be further inferred. Combining the two results, the final refinement direction information can be determined.

#### Final layer

The final layer can estimate the velocity of the object by calculating the spiking signals it receives from track recognition layer in a fix time interval. If in that interval, it receives many spiking signals from the previous layer, then it could conclude that the object is moving very slow, otherwise, it could conclude it’s moving fast. In addition, for the sake of preventing the object in the field of view within the ring movement, which may lead to the speed misjudgment, in this paper, the different small field of visions are divided according to the size of the sensor field of view. When the object moves out of the current small field area, the current decision is ended by the area judgment function, and then into the next small field of view of the judge.

### Optimization

**Fig 8:**
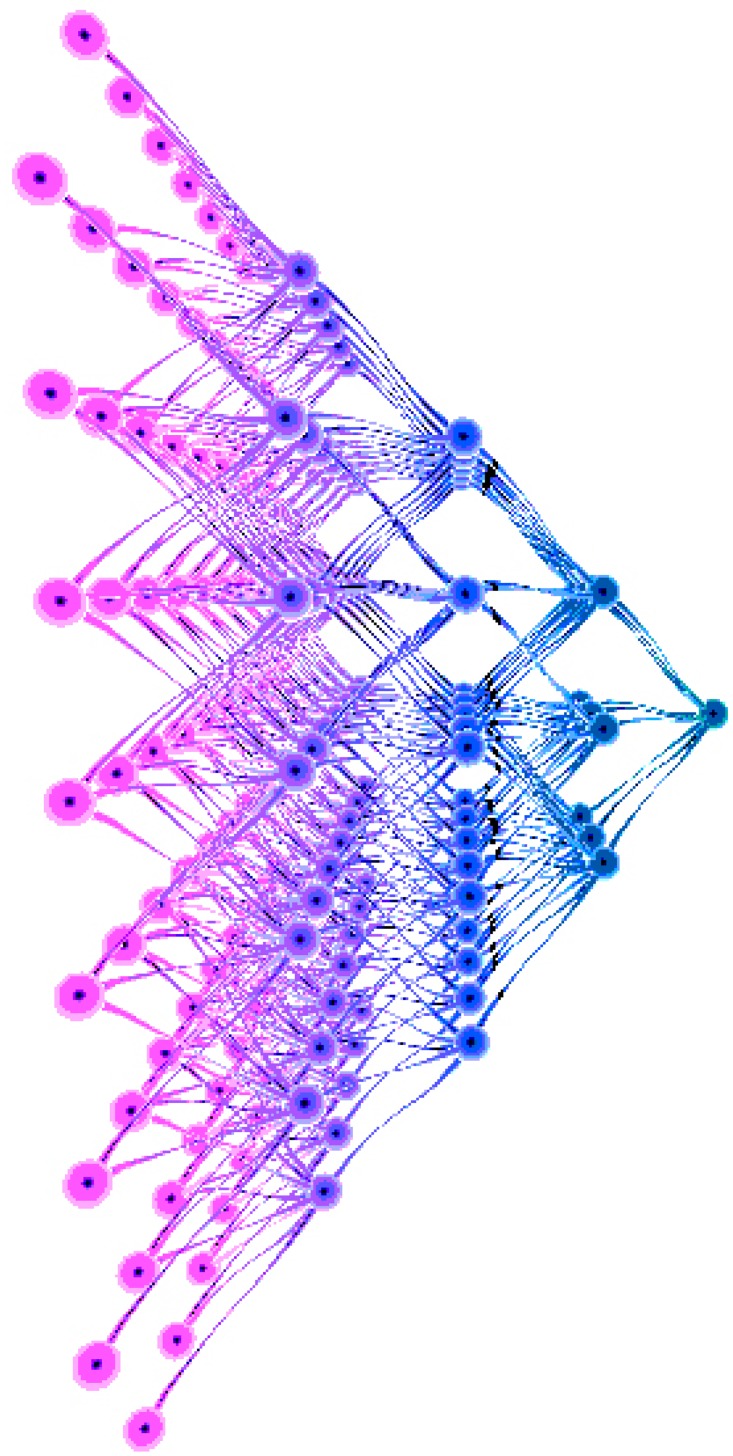
The bionic artificial neural network.

After module design, we have run an optimization phase to remove any unnecessary connections or structures by a pruning method. Fig 8 shows the final structure of our bionic artificial neural network built to imitate the functionality of Drosophila’s visual neural network. The neurons in the previous four layers are grouped in an 8*8 array. Every neuron in the second layer connects to 9 neurons from layer 1. Every neuron in layer 3 connects to 5 neurons in layer 2. Every neuron in layer 4 connects to 9 neurons in layer 3. There is only one neuron in layer 5, which connects to all the neurons in layer 4. The total scale of this neural network could be adjusted according to the requirements.

### Verification

In order to simulate and verify this bionic artificial neural network, we have built a simulation environment following CPU+GPU style. CPU is in charge of control, while GPU is used for computation for each neuron. The GPU we used is Nvidia Quadro K2200. In our simulation environment, a lot of parameters could be configured, such as the neuron model, connection between neurons, network scale, and so on.

**Fig 9:**
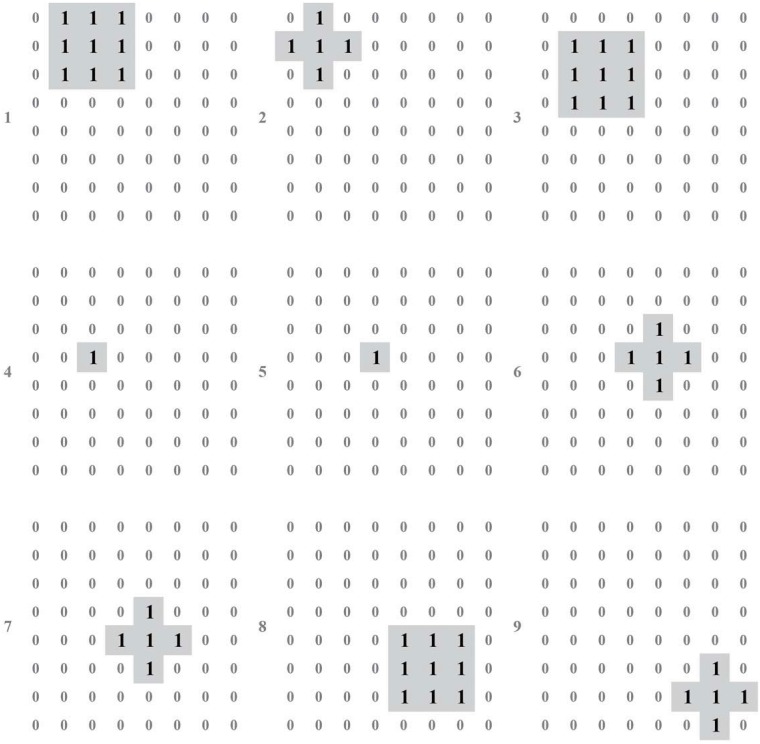
Output of layer 1.

**Fig 10:**
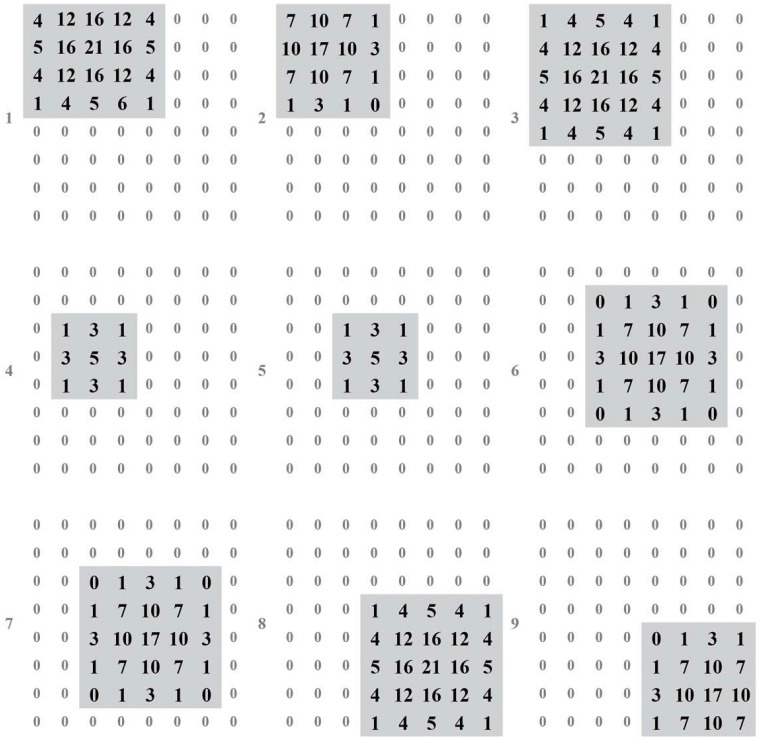
Output of layer 2.

**Fig 11:**
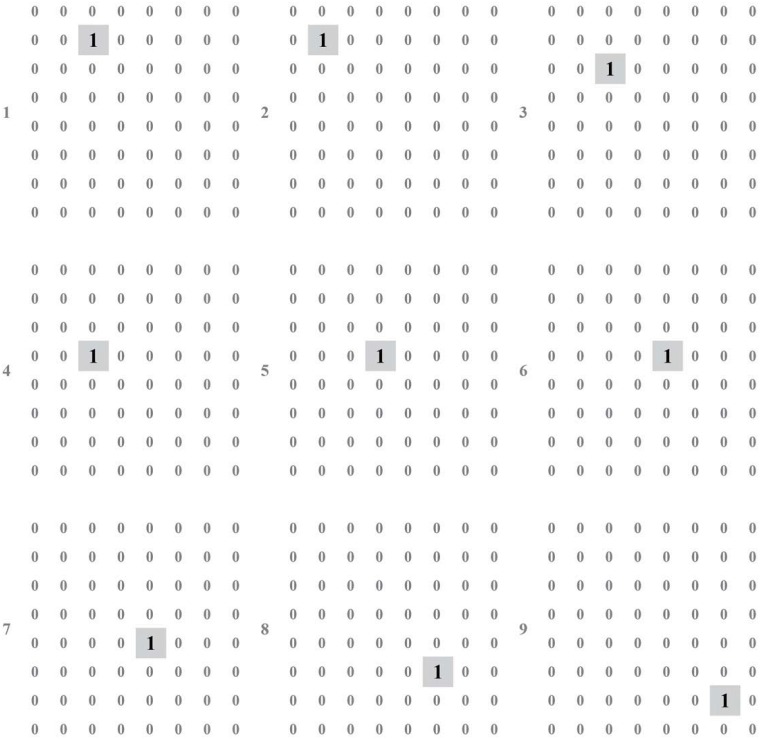
Output of layer 3.

**Fig 12:**
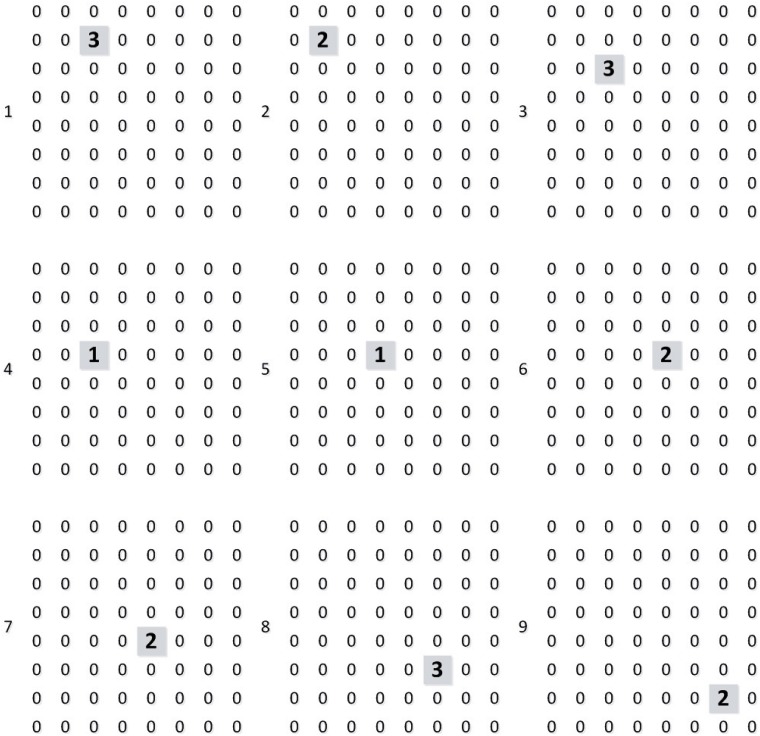
Output of distance.

**Fig 13:**
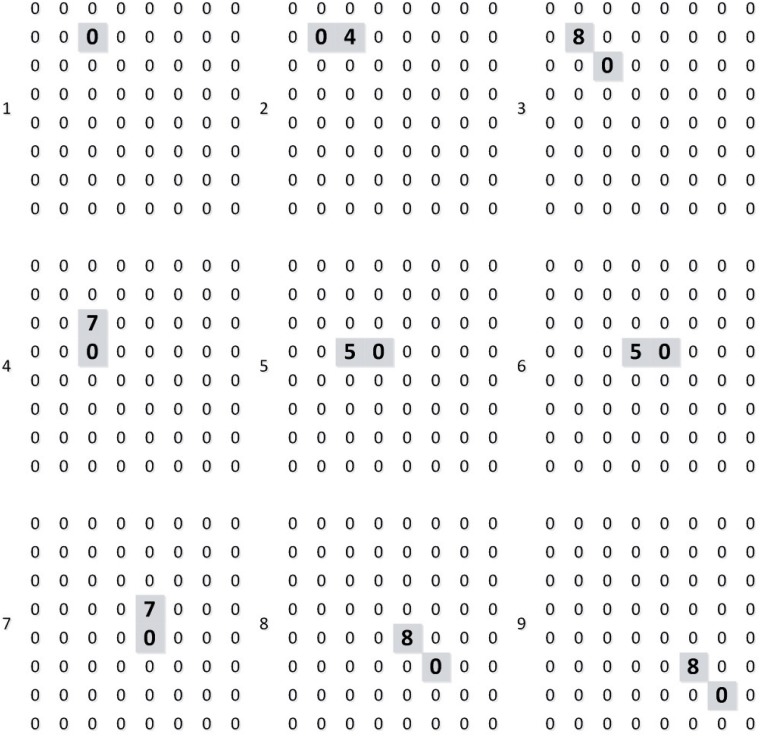
Output of layer 4.

We build the test environment as an imitation track of a moving object. And Fig 9 shows the output of layer 1 (perception layer) for 9 continuous time stamps. This data is feed to layer 2 (range estimation layer) and generates the output shown in Fig 10, which is the weighted sum of the output of neurons in layer 1. Then, location identification layer identifies the location of that object by comparing the sum results from layer 2, as shown in Fig 11. The neuron in layer 3 will send a spiking signal to tell neuron in layer 4 about the current location to form the track for that object and help to decide the moving direction. Also, according to the output of layer 2 and layer 3, the distance of that moving object is also generated, as shown in Fig 12. Here the number is the distance judged by the neural network (3 means faraway, while 1 means near). According to our design, the spiking output from layer 3 will indicate the current location to layer 4, thus layer 4 could output the moving direction of that object, as shown in Fig 13. In Fig 13, current location of the moving object is denoted as a “0“, while the moving direction is denoted as a number at previous location of that object. The meaning of the number for the moving direction is shown in Fig 7. The single neuron in layer 5 will decide the approximate velocity of that moving object by counting how many signals it received during a fix time interval. Through the comparison of Figs 9 to 13, it could be concluded that our neuron network can find the correct location, distance, and moving direction of that moving object.

## Contrastive analysis with actual compound eyes

The bionic artificial neural network designed in this paper has the function of detecting the velocity, direction, and distance of the object, viewed as a bionic compound eye, as is the actual Drosophila compound eye. The comparison of biological and this bionic compound eye [19–23] is shown in Table 1. Due to the lack of Drosophila compound eye data, the first three cited data are from bees, except the last one. It can be found that, in terms of observation distance, the perceived perspective of a single small eye in this subject increases by 30 degrees, resulting in an approximately two-fold increase in observable distance. From the reaction time, the biological compound eye needs 10ms for minimum processing of information, and ours is 100 times faster. From the fastest resolution, when the object in the field of vision stays less than 1ms, the biological compound eye can’t feel the presence of objects, while the efficiency of the bionic compound eye is close to 100 times. Moreover, judging from the accuracy of direction, there are only four kinds of direction determining cells in Drosophila, which can only be judged in four directions: upper, lower, left and right, while our work will be refined to eight directions.

**Table 1:** Comparison of biological and bionic compound eye.

Parameter(compound eye)	Biological	Bionic
Sight Distance	About 1 meter	About 2.2 meter
Reaction Time	10ms	0.1ms
Fastest Resolution	1ms	0.013ms
Directional Accuracy	4 groups	8 groups

Yet, The biological compound eye also has a detection function on the edge of the object, our bionic eye does not currently have this function, which is the next stage of optimization.

## Conclusion

In this work, we presented a method to implement artificial neural network through bionic construction rather through huge scale’s training and learning. This idea is inspired by the biological discoveries that, biological neural network could be implemented through two means: by congenital heredity, or by posteriority learning. The artificial neural network implemented through construing-type neural network could be used to perform some specific tasks, would have a more compact and concise structure. The case study verifies the validation of our method, and shows that our neural network can imitate the function of Drosophila’s visual neural network with similar features.

This is the first step toward an automatic process to generate the bionic artificial neural network. The construction of this neural network is conducted manually in this work. Later, we aim to use our simulation platform to find out sequences of environment inputs to promote the bionic neural network to auto-generate into the target neural network we presented in this work. And we are already adopting this method to build neural network to accomplish moving object detection and tracking. It’s a very demanding application nowadays.
